# Systematic Review: Exploring the Effectiveness of Health Care Competency Training as a Means of Suicide Prevention in LGBTQ+ Youth

**DOI:** 10.1016/j.jaacop.2025.06.010

**Published:** 2025-07-07

**Authors:** Kelly Cembrale, Ioannis Demopoulos, Angelica Terepka, Leonell Torres-Pagán, John Usseglio

**Affiliations:** a Saint George’s University, West Indies, Grenada; b NYC Health + Hospitals/Elmhurst, Queens, New York; c Private Practice, New York, New York; d Columbia University, New York, New York

**Keywords:** culturally competent care and training, health care workers, LGBTQ+ population

## Abstract

**Objective:**

To evaluate the effectiveness of LGBTQ+ competency training programs for health care professionals and examine the impact of these programs on LGBTQ+ youth suicidal thoughts, behavior, attempts, and risk.

**Method:**

This systematic review included quantitative studies that assessed LGBTQ+ cultural competency. Searches of 6 electronic databases yielded 6,317 records and 77 grey literature records (last searched on May 20, 2024); 19 studies met the inclusion criteria. Risk of bias was assessed using Cochrane ROB 2.0 and the Cochrane Robins I tool. Data on study design, sample characteristics, training details, and outcomes were extracted in line with the Cochrane Handbook and Preferred Reporting Items for Systemic Reviews and Meta-Analyses (PRISMA) guidelines.

**Results:**

Included studies varied: 13 mixed methods, 4 quasi-experimental, 1 randomized controlled trial, and 1 time series. All studies were conducted in Western countries and involved health care professionals, with varying training formats and durations. Training programs generally improved objective measures of knowledge, skills, and clinical preparedness. However, self-perceived knowledge, attitudes, and comfort levels showed inconsistencies. Only 6 studies addressed health care workers’ knowledge of LGBTQ+ youth suicide risk, focusing on general cultural competency rather than suicide risk reduction. None of the studies examined suicide prevention outcomes.

**Conclusion:**

Although LGBTQ+ cultural competency training shows improvements in health care professionals’ competence, studies are limited by methodological flaws, including reliance on self-assessments, small sample sizes, limited long-term data, high attrition rates, and variable assessment tools. A gap exists in understanding the impact of training on LGBTQ+ youth suicide risk. Future research should explore the association between health care provider cultural competency training and long-term suicide prevention outcomes.

**Study registration information:**

Ask for their pronouns, save their life: Exploring the effectiveness of training programs on healthcare competency as a means of suicide prevention in LGBTQ+ youth: A systematic review; https://www.crd.york.ac.uk/PROSPERO/view/CRD42024529991

**Diversity & Inclusion Statement:**

One or more of the authors of this paper self-identifies as a member of one or more historically underrepresented racial and/or ethnic groups in science. One or more of the authors of this paper self-identifies as a member of one or more historically underrepresented sexual and/or gender groups in science.

## Plain language summary

The authors conducted a systematic review of 19 studies on LGBTQ+ cultural competency training for healthcare professionals to evaluate its potential impact on LGBTQ+ youth suicide risk. Most studies found that training improved providers’ knowledge and confidence in working with LGBTQ+ youth. However, only 6 studies specifically addressed workers’ knowledge about LGBTQ+ youth suicidal thoughts, behaviors, attempts, and risks. None of the studies examined whether the cultural competency training influenced outcomes related to suicidal thoughts or behaviors. The findings show a need for more research to understand how this type of training could reduce suicide risk among LGBTQ+ youth.

The LGBTQ+ community is diverse, encompassing people from various races, ethnicities, ages, socioeconomic backgrounds, and global origins. The acronym LGBTQ+ stands for lesbian, gay, bisexual, transgender, and queer/questioning, with the plus (+) symbolizing inclusivity for other sexual orientations or gender identities. Recent studies show an increase in LGBTQ+ identification among youth, with increasing proportions of younger generations identifying as LGBTQ+.^1^, ^2^, ^3^

Despite advancements in LGBTQ+ rights and societal acceptance, LGBTQ+ youth continue to face considerable health disparities. LGBTQ+ youth are disproportionally affected by discrimination, harassment, and bullying—factors strongly associated with higher rates of suicidal ideation and attempts.^4^ According to a report from the US Centers for Disease Control and Prevention published in 2022,^5^ suicide was the second leading cause of death among youth. LGBTQ+ youth attempt suicide at a rate 4 times that of their cisgender heterosexual counterparts.^6^ The Trevor Project^7^ reported in 2024 that 41% of LGBTQ+ youth (ages 13-24) in the United States considered attempting suicide in the previous year, and 14% attempted suicide in the previous year. These numbers look even more stark for gender nonconforming and trans youth, who experience depressive symptoms, suicidal thoughts, and suicide attempts at rates 2 to 2½ times higher than cisgender LGBTQ+ youth.^7^ These statistics highlight the severe mental health crisis faced by LGBTQ+ youth, exacerbated by social rejection, discrimination, and stigma.^4^

However, it is important to recognize the existence of protective factors at both individual and community levels that can help prevent suicidal behaviors, ideation, and attempts among LGBTQ+ youth. Supportive communities, affirming familial and peer relationships, and inclusive school policies have been shown to mitigate the adverse effects of societal stigma.^8^ Mental health interventions specifically tailored to LGBTQ+ youth, including LGBTQ+ affirming counseling and therapy, can play a significant role in suicide prevention.^9^^,^^10^

Despite increased awareness, LGBTQ+ youth struggle to access mental health support tailored to their needs owing to insufficient culturally competent and affirming care.^11^^,^^12^ Many health care providers receive minimal training on LGBTQ+ cultural competency, including nonclinical staff (eg, front desk staff), who are often the first point of contact.^13^ This lack of training can impact patient care quality. For instance, only 16.7% of LGBTQ+ adolescents reported their pediatrician inquired about their sexual orientation,^14^ and many providers lack knowledge on critical topics such as gender-affirming surgery, hormone therapy, puberty blockers, gender dysphoria, pre-exposure prophylaxis, community LGBTQ resources, homelessness, and environmental risks.^15^ As a result, LGBTQ+ youth often feel frustrated and end up educating their health care providers themselves.^16^

In addition to a lack of provider competency, research has found that LGBTQ+ youth experience higher stigma and discrimination in health care settings than their peers.^16^ Research shows that 1 in 5 LGBTQ+ patients face discrimination in health care settings.^17^ Additionally, LGBTQ+ individuals are more likely to be denied essential medications and experience verbal or physical violence during physical examinations, leading them to avoid care.^18^ These experiences discourage LGBTQ+ youth from seeking care, as LGBTQ+ individuals are 3 times more likely than heteronormative peers to report avoiding medical care in the past year because of discrimination from health care providers.^19^

Cultural competency in health care for LGBTQ+ youth involves assessing the knowledge,^13^^,^^20^, ^21^, ^22^, ^23^, ^24^, ^25^, ^26^, ^27^, ^28^, ^29^, ^30^, ^31^, ^32^, ^33^, ^34^ attitudes/awareness,^13^^,^^21^, ^22^, ^23^^,^^25^, ^26^, ^27^^,^^31^^,^^32^^,^^34^ skills,^21^^,^^27^^,^^34^, ^35^, ^36^ behaviors,^37^ self-efficacy,^30^^,^^34^^,^^35^ clinical preparedness or confidence,^20^^,^^22^^,^^25^^,^^35^^,^^37^ and comfort of health care professionals in providing care.^20^^,^^24^^,^^33^ Cultural competency necessitates understanding social and cultural factors that influence patients’ health and applying skills that engage patients openly and inclusively.^38^^,^^39^ Health care providers must meet the evolving needs of the LGBTQ+ community, ensuring quality care at both individual and structural levels.^40^^,^^41^ Effective training enables providers to navigate the challenges faced by LGBTQ+ patients, providing equitable, compassionate care that respects sexual orientation, gender identity, and lived experiences.^39^^,^^41^ This fosters an environment where LGBTQ+ individuals feel safe, valued, and confident in seeking care.

Health care providers working with LGBTQ+ youth must understand gender and sexual identity development, the stigma faced by gender and sexual minorities, and the impact of social and family rejection on mental health, including suicide.^8^^,^^42^^,^^43^ Providers must recognize how harmful experiences both within and outside health care settings, such as discrimination, microaggressions, and invalidation, can decrease health-seeking behavior and increase the risk of suicidal behavior.^4^^,^^44^ Providers can create a welcoming space by expressing openness and normalizing gender and sexual diversity, ensuring that practices, policies, and language promote inclusivity and respect for all, and referring patients to specialists for gender-affirming care such as hormone replacement therapy or puberty blockers.^45^^,^^46^ Access to these services is critical for supporting transgender youth and preventing suicide.^47^^,^^48^

Cultural competency differs for lesbian, gay, or bisexual and transgender individuals. For lesbian, gay, or bisexual individuals, cultural competency focuses on sexual orientation, disclosure, and the impact of homophobia and bisexual erasure.^49^ For transgender individuals, it emphasizes gender identity and expression, the use of preferred pronouns, and understanding unique health care needs such as hormone replacement therapy and gender- affirming surgeries.^45^^,^^46^ Many cultural competency programs combine these areas into a single training, addressing sexual orientation and gender identity.

The implementation of LGBTQ+ cultural competency training remains inconsistent. The 2022 Healthcare Equality Index,^50^ a national LGBTQ+ benchmarking tool, found that although more institutions are pursuing accreditation, only 55% (486 institutions) qualify as LGBTQ+ Healthcare Equality Leaders, mostly in academic medical centers or in the Western and Northeastern United States. As of 2025, it is unclear how many states and territories in the United States have implemented cultural competency training for health care professionals as a requirement for license renewals, indicating a significant gap in policy and practice.

Studies have shown that health care professionals with higher levels of LGBTQ+ competency are better able to create supportive, affirming environments, use inclusive language, and offer appropriate care for gender and sexual minority youth.^51^ A previous systematic review broadly evaluated LGBTQ+ cultural competency among health care professionals,^41^ but did not specifically address LGBTQ+ youth. Given the increased prevalence of suicidal thoughts and behaviors, including suicide attempts, among LGBTQ+ youth compared with their heterosexual peers,^52^^,^^53^ it is critical to evaluate the effectiveness of cultural competency training tailored for health care professionals working with this population.

This systematic review aimed to evaluate the impact of cultural competency training programs for health care professionals in addressing the needs of LGBTQ+ youth. It also examined how improving the LGBTQ+ cultural competency of health care providers can help reduce suicidal thoughts, behavior, attempts, and risk and improve overall mental health outcomes in LGBTQ+ youth. Finally, the review explored the need for new strategies to improve the well-being and health care experiences of LGBTQ+ youth.

## Method

### Design

This systematic review was conducted according to the guidance from the *Cochrane Handbook for Systematic Reviews of Interventions*^54^ and reported in compliance with PRISMA 2020 checklist^55^ for proper reporting and was registered with PROSPERO. The review was conducted between March 2024 and July 2024. A meta-analysis was not performed because the necessary data were not consistently reported across the included studies. Specifically, required statistical data related to LGBTQ+ youth cultural competency and suicide-related outcomes (eg, effect sizes and specific measures of suicide risk, behaviors, and attitudes) were missing, which led to the decision to proceed with a qualitative narrative synthesis instead.

### Search Strategy

In collaboration with an academic librarian, relevant literature on LGBTQ+ youth cultural competency training for health care professionals was collected from 6 electronic databases: American Psychological Association PsycINFO (EBSCO), CINAHL (EBSCO), CENTRAL (Cochrane Central Register of Controlled Trials), Embase (Elsevier), ProQuest (Clarivate), and PubMed (National Library of Medicine). Ongoing clinical trials were searched for via ClinicalTrials.gov and International Clinical Trials Registry Platform (World Health Organization). Grey literature was included in the search process to provide thorough coverage of relevant sources. Search logic was constructed by integrating terms associated with adolescents, LGBTQ+ population, health care workers, culturally competent care, and training.

An example of the search strategies used in the databases is shown in Table S1, available online. All references were exported and managed using Covidence systematic review software,^56^ with duplicates removed. All searches were from inception until the date of search, with no filters applied. All electronic database searches were run on May 20, 2024. In addition to searching the databases, reference lists of studies included in the systematic review were reviewed to identify additional studies to include for screening. The reference lists of relevant systematic reviews and meta-analyses picked up by the electronic database search were reviewed to identify additional studies to include for screening. Lastly, tables of contents of selected journals in the field were searched for additional studies to include for screening. No limit on publication date was set.

### Inclusion and Exclusion Criteria

Articles were included in the review if LGBTQ+ youth from 13 to 24 years old comprised 100% of the sample; the effectiveness of LGBTQ+ cultural competency trainings was evaluated; one or more outcomes of trainings (eg, change in knowledge, skills, or attitudes) were quantitatively measured; institutionalized and non-institutionalized health professionals in any discipline (eg, physicians, nurses, social workers, psychologists, community health care workers, school health care workers, residents, medical students) were included; and the article was written in English. In addition to peer-reviewed articles, grey literature and dissertations were included in the review because of their relevance and contribution to the topic, particularly when peer-reviewed sources were limited. Grey literature was searched, and dissertations were included, but study protocols and conference abstracts were excluded from the review. Only published, peer-reviewed articles and full reports were considered for inclusion.

All health care settings, including hospitals, specialized clinics, school-based health centers, and others serving LGBTQ+ youth, were considered. The research settings were unrestricted, allowing interventions or studies to be conducted in free-living conditions, laboratory settings, hospital environments, or combinations of these.

Geographic limitations were not imposed in the review. Whereas health care systems and medical training models vary across countries, the experiences and needs of LGBTQ+ youth are not confined to specific geographic regions. Including studies from diverse countries may facilitate the identification of common themes and best practices in cultural competency training for LGBTQ+ youth, applicable across global health care settings.

### Study Design Eligibility

Eligible study designs included randomized controlled trials (RCTs), nonrandomized controlled trials, cohort studies, and other nonrandomized designs that quantitatively assessed the effectiveness of LGBTQ+ youth cultural competency training.

### Selection Process

Studies were screened in a 2-stage process using Covidence.^56^ Two independent reviewers screened each record title and abstract. Any disagreements were resolved by a third independent reviewer. The remaining records underwent full-text eligibility screening by 2 independent reviewers. At this stage, the screeners selected a reason from the exclusion criteria if they voted to exclude a record. Any disagreements were resolved by a third independent reviewer.

### Quality Appraisal

The Cochrane Risk of Bias 2.0 assessment tool^57^ for randomized controlled trials was used for the RCT studies. Criteria for risk of bias were categorized as low, high, or unclear based on guidelines from the Cochrane Handbook for Systematic Reviews of Interventions.^54^ For nonrandomized studies, the Cochrane ROBINS-I tool^58^ was used, evaluating risk of bias criteria as yes, partial yes, partial no, or no. To ensure consistency in risk of bias assessment, each record underwent critical appraisal by 2 independent reviewers using the respective risk of bias tool. Results from both assessments were reviewed by the team to finalize the risk of bias assessment for each record.

### Data Extraction (Selection and Coding)

Each study was extracted and organized into an evidence table using the matrix method. Two independent reviewers extracted and organized the data from each individual study separately. In cases of discrepancies between the 2 reviewers, a third independent reviewer resolved the differences through discussion and consensus. A third independent reviewer reviewed both tables to ensure accuracy. The following data from each record were extracted: country/region, study design, sample characteristics, training setting (eg, voluntary or mandatory), training (topic, modality, duration, trainer, source of material), measurement instrument, effect size, and key findings. These variables were edited based on the trialing of the data extraction template.

### Data Synthesis

This systematic review involved a narrative synthesis, and the analysis was operated at an aggregate level rather than at the level of individual participants. It evaluated the effectiveness of LGBTQ+ youth health care competency training across various related cultural competency domains and assessed their impact on addressing suicidal thoughts, behavior, attempts, and risk among LGBTQ+ youth.

## Results

A total of 6,317 citations were identified in electronic databases, and 77 articles were identified from grey literature. After removing duplicates, 4,616 unique abstracts remained. Following title and abstract screening, 4,535 abstracts were excluded as they did not align with the aim of this review.

**Figure 1:**
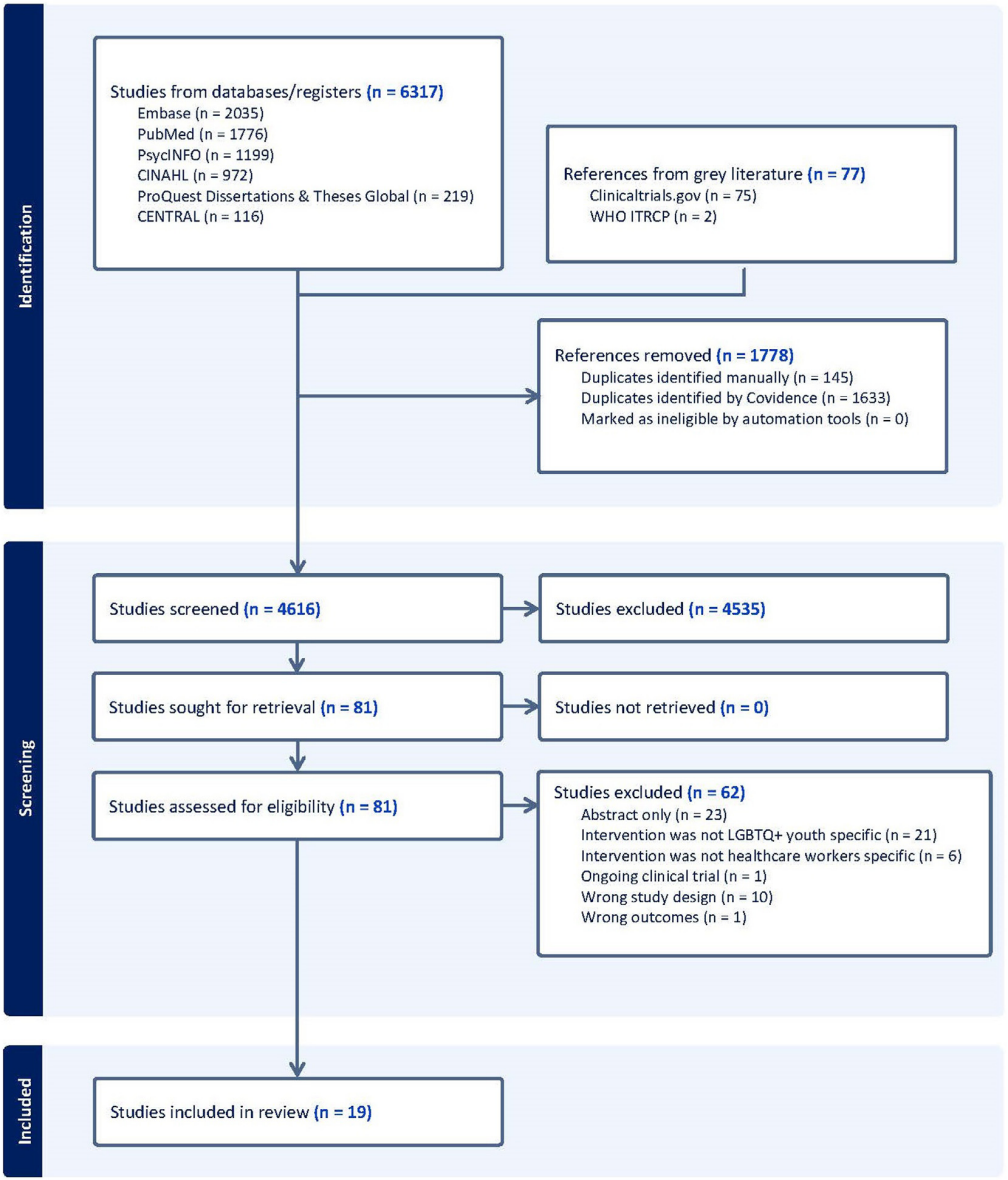
PRISMA Flow Diagram

Subsequently, 81 articles underwent full-text review, with 62 articles excluded for various reasons, such as having only abstracts, focusing on interventions specific to non-LGBTQ+ youth, involving non–health care worker participants, using incorrect study designs, being ongoing clinical trials, or using incorrect outcome measures. The remaining 19 articles met all criteria and were included in the review. The PRISMA flow diagram illustrating this process is shown in Figure 1.

Tables 1 and 2 summarize the 19 reviewed studies with 4 experimental designs: cohort study with a pretest/posttest (n = 13),^20^^,^^22^^,^^23^^,^^26^, ^27^, ^28^, ^29^, ^30^, ^31^, ^32^, ^33^^,^^35^^,^^36^ mixed methods (n = 4),^24^^,^^25^^,^^34^^,^^59^ RCT (n = 1),^21^ and time series (n = 1).^37^ Only 21.1% of the studies included follow-up measures to assess retention.^25^, ^26^, ^27^^,^^29^ All studies were conducted in Western countries: the United States (n = 15),^20^, ^21^, ^22^, ^23^, ^24^^,^^26^, ^27^, ^28^^,^^30^^,^^31^^,^^33^, ^34^, ^35^, ^36^, ^37^ Australia (n = 1),^59^ Ireland (n = 1),^25^ Italy (n = 1),^29^ and Switzerland (n = 1).^32^ Over the past 5 years, 10 studies were conducted.^22^^,^^24^^,^^25^^,^^28^^,^^29^^,^^31^^,^^35^, ^36^, ^37^^,^^59^ Sample sizes ranged from 18 to 169, with 36.8% involving small samples (n ≤ 30). The studies involved various health care professionals, including physicians and trainees (n = 11),^22^, ^23^, ^24^, ^25^^,^^28^, ^29^, ^30^, ^31^, ^32^^,^^35^^,^^37^ medical students (n = 8),^20^^,^^23^^,^^28^^,^^30^, ^31^, ^32^^,^^35^^,^^37^ nurse practitioners or physician assistants and trainees (n = 6),^23^^,^^24^^,^^30^^,^^31^^,^^35^^,^^37^ nursing staff and trainees (n = 6),^22^^,^^24^^,^^25^^,^^33^^,^^36^^,^^37^ psychologists and trainees (n = 4),^26^^,^^27^^,^^37^^,^^59^ school counselors and trainees (n = 2),^21^^,^^34^ social workers (n = 3),^22^^,^^26^^,^^37^ school health staff (n = 3),^21^^,^^26^^,^^34^ and other health staff (n = 3).^24^^,^^33^^,^^37^

**Table 1:** Characteristics of Included Studies

Author, year	Country	Study design	Recruitment method	Sample	Training modality	Training topic
Bakhai, *et al.* 2016^20^	The United States of America	Pretest/posttest	Unclear	N=42 **Participants**: Third- and fourth-year medical students **Response Rate**: 95% **Attrition**: 3%	**Multimodal**: Flipped classroom, small group learning, and peer-to-peer instruction	**Population focus**: LGBTQ+ youth **Topics**: Focusing on affirming SGM youth identities, enhancing family communication, fostering interdisciplinary collaboration, addressing mental health challenges, and establishing safe clinical environments for SGM adolescents. **Trainer**: Two adolescent medicine faculty members with expertise in SGM health and one pediatric clerkship director. **Duration**: A single 2-hour session divided into four sections. 1st section: 15 min, 2nd section: 30 min, 3rd section: 50 min, 4th section: 25 min **Source of Training**: Not applicable
Byrd, *et al.* 2013^21^	The United States of America	RCT	Convenience Sampling	N=77 **Participants**: School counselor trainees and school counselors **Response Rate**: 100% **Attrition**: 4%	**Multimodal**: Discussions, roleplaying exercises, case studies and hand-outs	**Population focus**: LGBTQ+ youth **Topics**: Modifying non-affirmative school environments, strategies for intervening in homophobic responses at school, emphasizing school counselors' role in educating staff about issues affecting sexual minorities. **Trainer**: Doctoral student **Duration**: One 3-h session for experimental group; unclear control group **Source of Training**: GLSEN Safe Space
Goodall, *et al.* 2024^22^	The United States of America	Pretest/posttest	Voluntary	N=25 **Participants**: Physician, staff, nurse, nursing assistant, and social worker **Response Rate**: 100% **Attrition**: 12%	**Multimodal**: In-person module, e-Learning module, educational resources, and role-playing scenarios of a transgender youth coming in for suicidal ideation	**Population focus**: LGBTQ+ youth **Topics**: Cultural competence in healthcare using Campinha-Bacote’s model, learning about healthcare for LGBTQIA+ youth, gaining practical experience with transgender patients through role-playing, and applying family-centered care strategies in a pediatric emergency department. **Trainer**: Interdisciplinary team of stakeholders (a bedside nurse and DNP student, a nurse manager, an academic faculty member, and the hospital’s director of LGBTQ + Health) **Duration**: One 1-h session **Source of Training**: Josepha Campinha-Bacote’s Model
Hodax, *et al.* 2024^37^	The United States of America	Time Series	Voluntary	N=119 (Series 1 N=58; Series 2 N=61) **Participants**: Doctors naturopathic medicine providers, advanced practice provider, residents, medical students or health professionals, psychologist, social workers, nurses, medical assistants, dental assistants/dental clinic managers, educators, family/patient services specialist, registered dietitian, speech-language pathologist **Response Rate**: Unclear **Attrition**: Unclear	**Online/virtual**: Didactic presentation and case discussions	**Population focus**: Transgender and non-binary youth **Series 1 Topics**: Introduction to gender diversity, affirming clinical environment, supporting families and schools, mental health, social transition, and eating disorders and autism. **Series 2 Topics**: Introduction and consent, gender dysphoria and euphoria, puberty blockers, masculinizing treatments, feminizing treatments, and gender-affirming surgery. **Trainer**: Multidisciplinary team of medical and mental health providers from the Seattle Children’s Gender Clinic. Case presentations were presented by ECHO participants. **Duration**: Two series of six monthly sessions each, with each session lasting 60 minutes for Series 1 and each session lasting 75 minutes for Series 2. **Source of Training**: Project ECHO
Keenhold. 2022^24^	The United States of America	Mixed Methods (QUANTI-quali)	Convenience Sampling	N=30 **Participants**: Medical doctors, doctors of osteopathy, registered nurses, medical assistants, and psychiatric mental health nurse practitioners **Response Rate**: 40% **Attrition**: 25%	**Online module**: PowerPoint presentation	**Population focus**: LGBTQ+ youth **Topics**: Understanding the needs of LGBTQ+ adolescents, cultural competence in care provision, terminology and language sensitivity, evidence-based practices, affirming communication skills, and addressing mental health disparities. **Trainer**: Not applicable **Duration**: Estimated duration of one session, lasting 20 minutes. **Source of Training**: Not applicable
Kelleher, *et al.* 2023^25^	Ireland	Mixed Methods (QUANTI-quali) Follow-up (12 weeks)	Voluntary	N=40 **Participants**: Doctors and nurses **Response Rate**: 80% **Attrition**: 44%	**Online/virtual module**	**Population focus:** LGBTQ+ youth **Topics:** Not reported. **Trainer:** Not applicable **Duration:** A single 1-h session **Source of Training:** Not applicable
Luke, *et al.* 2017 34	The United States of America	Mixed Methods (Sequential-quali)	Mandatory	N=24 **Participants**: School counseling trainees **Response Rate**: 100% **Attrition**: 42%	**In-person:** Lectures, discussions, reading articles, and in-person experience co-facilitating LGBTQ+ student groups	**Population focus:** LGBTQ+ youth **Topics:** Experiences of LGBTQ+ students, video clips of LGBTQ+ students telling their own stories, and debriefings/skill learning of co-facilitating with course facilitator and doctoral student assistant. **Trainer**: Course instructor and doctoral student assistant **Duration:** One session per week for 16 weeks, with trainees cofacilitating 3 to 6 LGBTQ student group meetings over two months and participating in one hour of group supervision after each session. **Source of Training:** Not applicable
McGravey. 2015^26^	The United States of America	Pretest/posttest Follow-up (6 weeks)	Voluntary	N=68 **Participants**: School psychologists, school adjustment counselors, guidance counselors, and school social workers **Response Rate**: 100% **Attrition**: 66%	**In-person**: Workshop of four modules	**Population focus:** LGBTQ+ youth **Topics:** Laws, demographics, identity development issues for LGBTQ+ youth; risk factors; and techniques for creating safer schools for LGBTQ+ youth. **Trainer:** Unclear **Duration:** One single session for all four modules, with a total duration of four hours, and each module was estimated to last 45-60 minutes. **Source of Training:** Not applicable
Nielsen. 2017^27^	The United States of America	Pretest/posttest Follow-up (2 months)	Voluntary	N=25 **Participants**: Psychology graduate students **Response Rate**: 44% **Attrition**: 50%	**In-person**: PowerPoint presentation with handouts and adapted Safe Space Training	**Population focus**: LGBTQ+ youth **First Training Topics**: Demographics, developmental issues, and risk factors for LGBTQ+ individuals. **Second Training Topics**: Safe Space training covers various topics such as pronouns, intersectionality, LGBTQ+ representation in media, respectful language, LGBTQ+ terminology, understanding LGBTQ+ issues, and being an ally. **Trainer**: Workshop (researcher); Safe Space Training (trained faculty member) **Duration**: The training consisted of two in-person sessions, each lasting 30 minutes and held one month apart. The second in-person session was followed by a Safe Space training session on the same day, lasting 45-60 minutes. **Source of Training**: Knowledge questions (McGravey 2014) and Safe Space Training
Riggs. 2021^59^	Australia	Mixed Methods (QUANTI-quali)	Voluntary	N=74 **Participants**: Psychologists **Response Rate**: 81% **Attrition**: 92%	**Multimodal**: Online/virtual module with a live author for questions	**Population focus**: Transgender and nonbinary youth. **Topics**: Ansara’s Cisgenderism framework outlines misgendering, binarizing, and pathologizing; TGNB demographics and definitions; pathways to care for TGNB youth; historical barriers and clinician gatekeeping in clinical care; DSM-5 criteria for gender dysphoria diagnosis; mental health disparities among TGNB youth; principles of gender-affirming care; case studies; the GENDER mnemonic for case formulation with TGNB youth and parents; and resources for clinicians and TGNB youth. **Trainer**: Psychotherapist specializing in working with TGNB youth **Duration**: Four 90-minute webinar sessions held over a two-month period. **Source of Training**: Australian Psychological Society Training Institute
Sandberg, *et al.* 2023^28^	The United States of America	Pretest/posttest	Voluntary	N=88 **Participants**: Attending physician, fellows, residents and medical students **Response Rate**: 48% **Attrition**: 50%	**Online module**	**Population focus**: Transgender and nonbinary youth **Topics**: Terminology, creating an inclusive environment, caring for nonbinary youth, ethical principles, managing gender diverse children before and during puberty (both medically and non-medically), medical care for adolescents going through puberty, surgical options, and addressing psychosocial aspects. **Trainer**: Not applicable **Duration**: Five modules, with an estimated total duration of two hours. Participants had 30 days to complete it. **Source of Training**: Developed by members of the Pediatric Endocrine Society's Transgender Special Interest Group (SIG)
Santamaria, *et al.* 2023^29^	Italy	Pretest/posttest Follow-up (3 months)	Voluntary	N=96 **Participants**: Pediatricians **Response Rate**: Unclear **Attrition**: Unclear	**In-person**: Theoretical and clinical practice sessions	**Population focus**: Transgender and nonbinary youth. **Theoretical practice**: Sexual identity foundations, developmental paths of gender identity in childhood and adolescence, minority stress impact, health disparities, guidelines for hypothalamic blockers in teens, and the role of family pediatricians in early support identification. **Clinical practice**: A case study of Giulio, a 13-year-old with gender dysphoria and had a facilitated group discussion on case management. **Trainer**: Experts in the field of gender diversity in youth **Duration**: A single 6-hour day, with two sessions. **Source of Training**: Not applicable
Vance, *et al.* 2017^23^	The United States of America	Pretest/posttest	Voluntary	N=20 **Participants**: Fourth year medical students, pediatric interns, psychiatry interns and nurse practitioner students **Response Rate**: 95% **Attrition**: 5%	**Multimodal**: Online modules and an observational experience at a child and adolescent gender clinic	**Population focus**: Transgender and nonbinary youth **Online Module Topics**: Understanding gender diversity and development within the transgender community; gathering psychosocial histories from LGBTQ+ individuals, using the HEADSS assessment for LGBTQ+ youth, conducting sensitive pubertal stage evaluations and physical exams for transgender youth, exploring therapeutic supports and medications for transgender youth. **Observational Experience**: attended the gender clinic’s multidisciplinary conference and followed one patient interaction for each of the disciplines. **Trainer**: Not applicable **Duration**: Online: 90 min (6 modules each consisting of 15 min); In-person: One single day of a 5-h observational experience. **Source of Training**: Author developed Transgender Youth Curriculum
Vance, *et al.* 2018^30^	The United States of America	Pretest/posttest	Voluntary	N=31 **Participants**: Fourth year medical students, pediatric interns, psychiatry interns and nurse practitioner students **Response Rate**: Unclear **Attrition**: 10%	**Online modules**	**Population focus**: Transgender and nonbinary youth **Online Module Topics**: Understanding gender diversity and development within the transgender community; gathering psychosocial histories from LGBTQ+ individuals, using the HEADSS assessment for LGBTQ+ youth, conducting sensitive pubertal stage evaluations and physical exams for transgender youth, exploring therapeutic supports and medications for transgender youth. **Trainer**: multidisciplinary staff including physicians and a psychologist **Duration**: One day of 6 modules lasting 15 min per session. **Source of Training**: Not applicable
Vance, *et al.* 2020^31^	The United States of America	Pretest/posttest	Unclear	Phase 1 N=20 **Participants**: Fourth year medical students, pediatric interns, psychiatry interns, and nurse practitioner students **Response Rate**: Unclear **Attrition**: Unclear	**Multimodal**: Online module and an observational experience at a transgender pediatric clinic	**Population focus**: Transgender and nonbinary youth **Online Module Topics**: Understanding gender diversity and development within the transgender community; gathering psychosocial histories from LGBTQ+ individuals, using the HEADSS assessment for LGBTQ+ youth, conducting sensitive pubertal stage evaluations and physical exams for transgender youth, exploring therapeutic supports and medications for transgender youth. **Observational Experience**: attended the gender clinic’ multidisciplinary conference and shadowed a doctor on patient visits. **Trainer**: multidisciplinary staff including physicians and a psychologist **Duration**: 1-h 17 min for online modules and followed by an afternoon session for observational experience on a different day. **Source of Training**: Not applicable
Vance, *et al.* 2021^35^	The United States of America	Pretest/posttest	Unclear	N=43 **Participants**: Fourth year medical students, pediatric interns, psychiatry interns and nurse practitioner students **Response Rate**: Unclear **Attrition**: Unclear	**Multimodal**: Online module with a video clinical vignette and standardized patient encounters	**Population focus**: Transgender and nonbinary youth **Online Module Topics**: Understanding gender diversity and development within the transgender community; gathering psychosocial histories from LGBTQ+ individuals, using the HEADSS assessment for LGBTQ+ youth, conducting sensitive pubertal stage evaluations and physical exams for transgender youth, exploring therapeutic supports and medications for transgender youth. **Standardized Patient Encounter (SPE)**: Practicing gender-affirming communication skills with transgender teenagers seeking hormone therapy through simulated patient encounters, using checklists to evaluate history-taking, counseling, and patient-centered communication. **Trainer**: multidisciplinary staff including physicians and a psychologist; actors portrayed the SPE patients after training by the faculty members **Duration**: 88-min for 6 online with video clinical vignette; 20 min for 2 SPE cases **Source of Training**: Not applicable
Wahlen, *et al.* 2017^32^	Switzerland	Pretest/posttest	Convenience Sampling	N=157 **Participants**: Fourth year medical students **Response Rate**: 68% **Attrition**: 40%	**In-person:** Lecture	**Population focus:** LGBT youth **Topics:** Health issues of LGBT adolescents. **Trainer:** Pediatrician **Duration:** A single 1-h lecture **Source of Training:** Not applicable
Walia, *et al.* 2019^33^	The United States of America	Pretest/posttest	Voluntary	N=169 **Participants**: Medical doctor faculty or trainee, registered nurse, certified registered nurse anesthetist, patient care assistants and surgical technicians **Response Rate**: 27% **Attrition**: Unclear	**In-person**: Didactic lecture and links to video recording provided for staff who missed lecture	**Population focus**: LGBT youth **Topics**: Differences between sexual orientation and gender identity, cultural competency, health disparities experienced by the LGBTQ community, culturally competent care for LGBTQ patients. **Trainer**: Director of the LGBTQ+ health initiative **Duration**: Unclear **Source of Training**: Not applicable
Wright - McGrath. 2023^36^	The United States of America	Pretest/posttest	Unclear	N=18 **Participants:** Nurses **Response Rate:** 100% **Attrition:** 0%	**Multimodal**: Offered online and In-person (PowerPoint presentation and educational material in packets)	**Population focus**: Transgender and nonbinary youth. **Topics**: Documenting preferred names and identified gender in the EMR, using preferred names and pronouns in patient interactions for gender-affirming care, creating educational materials on gender-affirming care protocols, and training clinical staff on proper documentation and providing gender-affirming care. **Trainer**: Nurse practitioner **Duration**: One single session of 30 min **Source of training**: Not applicable

Note: DNP = Doctor of Nursing Practice; EMR = electronic medical record; LGBT = lesbian, gay, bisexual, transgender; RCT = randomized controlled trial; SGM = sexual and gender minority; TGNB = transgender and nonbinary.

**Table 2:** Outcome Measures of Included Studies

Author, year	Measurement	Key findings	Effect size	Competency domains	Competency domains retention	Suicidality
Bakhai, *et al.* 2016^20^	Author developed survey. No report about reliability or validity.	Significant improvement in self - perceived comfort, preparedness, and knowledge for skills.		(+) Knowledge (+) Clinical preparedness (+) Comfort		
Byrd, *et al.* 2013^21^	Sexual Orientation Competency Scale (SOCCS), with reported reliability with Cronbach’s alpha of 0.84.^52^	A significant relationship existed between individuals who received the training and higher levels of knowledge, awareness, and skills.	Posttest control group administration: r^ˆ^2 = 0.38 Posttest intervention group administration: r^ˆ^2 = 0.48	(+) Knowledge (+) Awareness (Attitudes) (+) Skills		
Goodall, *et al.* 2024^22^	Lesbian, Gay, Bisexual, and Transgender Development of Clinical Skills Scale (LGBTDOCSS). No report about reliability or validity.^53^	After the 1-h session, there was a statistically significant increase in the clinical preparedness subscale.		(+) Clinical preparedness No change in attitudinal awareness and knowledge		
Hodax, *et al.* 2024^37^	Adapted from the TransInclusive Provider Scale. No report about reliability or validity.^60^	**Behavior Series 1:** Significant improvements from postsurvey compared to presurvey: introducing oneself to patients with name and pronouns; and asking patients what name and pronouns they use during every clinical encounter. **Confidence Series 1**: Significantly higher at postsurvey compared with presurvey for all items except for the providers’ abilities to ‘‘introduce myself to patients with my name and pronouns’’ and to ‘‘explain to a patient or family the difference between gender identity and sexual orientation.’’ **Confidence Series 2**: Significantly higher at midpoint and post-surveys’ compared with presurvey for all items.		(+) Confidence (+) Behavior		
Keenhold. 2022^24^	Author developed survey. No report about reliability or validity.	---		No change in objective knowledge, self-perceived knowledge, and comfort.		
Kelleher, *et al.* 2023^25^	Lesbian, Gay, Bisexual, and Transgender Development of Clinical Skills Scale (LGBTDOCSS). No report about reliability or validity.^53^	Compared with pretest, posttest competency scores increased significantly across all domains (knowledge, clinical preparedness, and attitudes) at 2 weeks and 12 weeks.		(+) Knowledge (+) Attitudes (+) Clinical preparedness	(+) Knowledge (+) Attitudes (+) Clinical preparedness	
Luke, *et al.* 2017^34^	Sexual Orientation Competency Scale (SOCCS), with reported reliability with Cronbach’s alpha of 0.84.^52^	SOCCS pretest to posttest indicated that trainees’ knowledge, skills, and school counseling–related self-efficacy with LGBTQ+ youth significantly increased, but trainees’ reported levels of awareness significantly decreased.	SOCCS Awareness: η2 = 0.23 SOCCS Knowledge: η2 = 0.78 SOCCS Skills: η2 = 0.55	(+) Knowledge (+) Skills (+) Self-efficacy (-) Attitudes		✔
McGravey. 2015^26^	Sexual Orientation Competency Scale (SOCCS);^52^^,^ with two or three author developed questions measuring self-perceived competency regarding transgender clients (no report or validity or reliability).	Significantly improved scores on SOCCS knowledge, SOCCS awareness, and Transgender SOCCS from pre-test to post-test. Significantly improved scores on SOCCS skills and transgender SOCCS from pre-test to follow-up. Significantly decreased scores on the SOCCS knowledge from post-test to follow-up.		(+) Knowledge (+) Awareness (Attitudes)	(+) Knowledge (+) Skills No change in awareness.	✔
Nielsen. 2017^27^	Sexual Orientation Competency Scale (SOCCS), with reported reliability with Cronbach’s alpha of 0.84.^52^	When compared to the pretest, the posttest showed significant changes in participants’ skills.		(+) Skills No changes to attitudes and knowledge.	No change in skills, attitudes and knowledge.	
Riggs. 2021^59^	Author-developed, with reported reliability with a Cronbach's alpha of 0.94.	From pretest to posttest, the confidence scores significantly increased.	Confidence *d* = 3.02	(+) Confidence		
Sandberg, *et al.* 2023^28^	Author developed survey. No report about reliability or validity.	After the 5-part module series, knowledge was significantly increased among providers in medical management for transgender and nonbinary youth in pubertal management and surgical interventions.		(+) Objective Knowledge		
Santamaria, *et al.* 2023^29^	Author developed survey. No report about reliability or validity.	Knowledge about sexual identity significantly improved from pre- to post-training and from pre- to follow-up assessment, but not from post-training to follow-up assessment.	Knowledge η2 = 0.46	(+) Objective Knowledge	(+) Objective Knowledge	
Vance, *et al.* 2017^23^	Unclear	Postcurriculum knowledge and awareness scores had a statistically significant increase on every queried transgender youth-related benchmark.		(+) Knowledge (+) Awareness (Attitudes) (+) Skills		✔
Vance, *et al.* 2018^30^	Author developed survey. No report about reliability or validity.	Comparing pre-and post- module evaluations, the learners’ median overall objective knowledge, scores, self-perceived knowledge, and self-efficacy scores significantly increased.		(+) Objective Knowledge (+) Subject Knowledge (+) Self-efficacy		✔
Vance, *et al.* 2020^31^	Author developed survey. No report about reliability or validity.	Phase 1: Statistically significant increase in self-perceived knowledge/awareness of transgender-related care.		(+) Knowledge (+) Awareness		
Vance, *et al.* 2021^35^	Author developed survey. Validity for standardized patient encounters was Cronbach alpha = 0.91, but no report about reliability or validity for assessments on the online modules/clinical vignette.	Overall self-efficacy scores increased significantly between pre-curriculum and post-curriculum and further increased between post-curriculum and post-standardized patient encounters. Comparison between the original curriculum with only online modules and the curriculum with added standardized patient encounter activities showed a statistically significant difference favoring the curriculum with standardized patient encounters in final self-efficacy scores.		(+) Self-efficacy (+) Skills		✔
Wahlen, *et al.* 2017^32^	Author developed survey. No report about reliability or validity.	Significant improvements in attitudes and knowledge scores from pre- to post-lecture among medical students.	Attitudes d = 0.14 Knowledge d = 0.84	(+) Objective Knowledge (+) Attitudes		✔
Walia, *et al.* 2019^33^	Author developed survey. No report about reliability or validity.	After training, there were no statistically significant improvements in self-perceived knowledge or comfort within the 6 domains. One provider had a perfect score for the objective knowledge before and after training while 14 had a perfect score after training, but not before. There was a statistically significant improvement in the proportion of providers with a perfect score after the training.		No change in objective knowledge, self-perceived knowledge and comfort.		
Wright - McGrath. 2023^36^	Author developed survey informed by WPATH. No report about reliability or validity.	------		No change in skills.		

Note: LGBT-DOCSS = Lesbian, Gay, Bisexual, and Transgender Development of Clinical Skills Scale; SOCCS = Sexual Orientation Counselor Competency Scale; WPATH = World Professional Association for Transgender Health.

Training programs varied in delivery format (in-person, online, multimodal) and duration (self-paced, instructor-led ranging from a single session lasting 20 minutes to 16 weekly sessions lasting 1 hour). Ten studies focused on LGBTQ+ youth broadly,^20^, ^21^, ^22^^,^^24^, ^25^, ^26^, ^27^^,^^32^^,^^33^^,^^35^ and 9 studies specifically addressed transgender and nonbinary youth.^23^^,^^28^, ^29^, ^30^, ^31^^,^^35^, ^36^, ^37^^,^^59^ Thirteen studies used instruments that did not include adequate requirements for statistical reliability and validity as they were designed by study authors without undergoing formal validation processes, and only 2 of these studies reported reliability measures, including Cronbach α, to assess the internal consistency of their measures.^35^^,^^59^ Validated instruments used included the Sexual Orientation Counselor Competency Scale (SOCCS)^61^ in 4 studies,^21^^,^^26^^,^^27^^,^^34^ and the LGBT-Development of Clinical Skills Scale (LGBT-DOCSS)^62^ in 2 studies.^22^^,^^24^ The SOCCS^61^ has been validated in terms of construct validity, demonstrated through factor analysis, as well as 1-week test-retest reliability. The LGBT-DOCSS^62^ has been validated in terms of construct validity, as demonstrated through exploratory and confirmatory factor analysis techniques, and 2-week test-retest reliability. The variability in study designs, participant groups, and instruments suggests that outcomes were inconsistent, indicating a need for further standardization to strengthen future research. The sample and training methods are summarized in Table 1.

**Figure 2:**
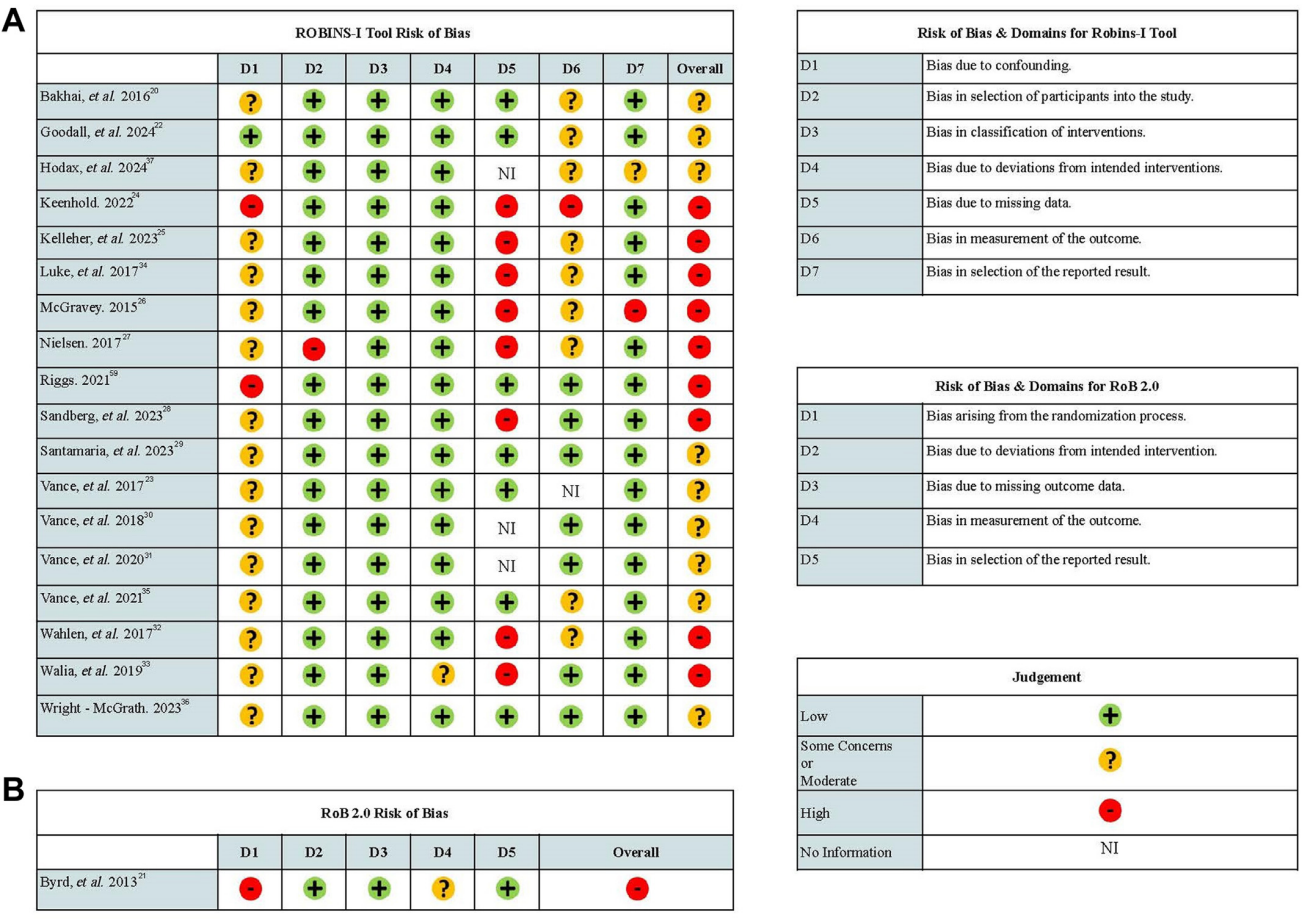
Quality Assessment ***Note:****Please note color figures are available online.*

The 19 studies meeting all criteria were assessed for quality and risk of bias (Figure 2). These studies included quasi-experimental studies and RCTs. Most studies showed moderate to high overall risk of bias for quasi-experimental studies and RCTs. Nonrandomized studies generally scored moderate to high on risk of bias, particularly in confounder control (eg, lack of sample controls, unclear recruitment methods), handling missing data (eg, low response rates, high attrition rates), and outcome measurement (eg, nonvalidated instruments, reliance on self-reports). One study flagged participant selection bias owing to recruitment from the same graduate program as the lead researcher.^27^ Most studies faced challenges because of small sample sizes, affecting the accuracy of the results. Another study demonstrated lower risk of bias across most domains except for outcome measurement, relying on self-reporting (eg, LGBT-DOCSS).^22^

The overall risk of bias for the RCT^21^ was determined to be high. Although the study had a robust design, including features such as convenience sampling, sensitivity analysis, a pretest/posttest design, low attrition, and use of a widely accepted training manual, there were significant limitations. Specifically, the authors did not adequately detail randomization procedures. It is unclear if enrollment was independent of the principal investigator before participant enrollment or if double blinding was used. Given these concerns, the study had a high overall risk of bias, despite showing low detection bias, as all participants received the training, and no deviations were reported.

Outcomes related to cultural competency, focusing on skills, attitudinal awareness, behaviors, and both objective and self-perceived knowledge, were examined in 18 studies.^20^, ^21^, ^22^, ^23^, ^24^, ^25^, ^26^, ^27^, ^28^, ^29^, ^30^, ^31^, ^32^, ^33^, ^34^, ^35^, ^36^, ^37^ Six studies evaluated health care workers’ skills from pretest to posttest after the intervention,^21^^,^^23^^,^^27^^,^^34^, ^35^, ^36^ with 5 of these showing a statistically significant increase.^21^^,^^23^^,^^27^^,^^34^^,^^35^ Furthermore, 1 study reported follow-up assessments showing no change in skills at 2-month follow-up.^27^ Additionally, 1 study reported a significant increase in skills from pretest to follow-up 4 to 6 weeks later, though it lacked pretest to posttest data.^26^ Nine studies assessed health care workers’ attitudinal awareness from pretest to posttest following the intervention.^21^, ^22^, ^23^, ^24^, ^25^, ^26^, ^27^^,^^31^^,^^32^^,^^34^ Regarding attitudinal awareness scores, 6 studies found a significant increase,^21^^,^^23^^,^^25^^,^^26^^,^^31^^,^^32^ and 1 study reported a significant decrease.^34^ Three studies included follow-up assessments^25^, ^26^, ^27^; however, only 1 study reported a significant increase in attitudinal awareness at follow-up.^25^ Additionally, 1 study examined health care worker behaviors, showing a significant improvement from pretest to posttest.^37^

Objective knowledge was assessed by 6 studies,^24^^,^^28^, ^29^, ^30^^,^^32^^,^^33^ with 4 reporting a statistically significant improvement in scores from pretest to posttest.^28^, ^29^, ^30^^,^^32^^,^^33^ Additionally, 1 study^29^ that assessed follow-up measures also found a significant increase in objective knowledge scores. Self-perceived knowledge for health care workers was evaluated by 12 studies from pretest to posttest,^20^, ^21^, ^22^, ^23^, ^24^, ^25^, ^26^, ^27^^,^^30^^,^^31^^,^^33^^,^^34^ and 8 studies reported a statistically significant increase in self-perceived knowledge after the intervention.^20^^,^^21^^,^^23^^,^^25^^,^^26^^,^^30^^,^^31^^,^^34^ Furthermore, 1 study reported an increased knowledge in follow-up measures.^25^ However, 1 study reported a significant decrease in self-perceived knowledge from posttest to follow-up.^26^

Some studies assessed outcomes related to self-perceived cultural competence, including clinical preparedness/confidence, self-efficacy, and comfort levels, rather than directly measuring cultural competence through testing providers’ knowledge or observer-rated evaluations of performance. Five studies reported a statistically significant increase in participants’ clinical preparedness and confidence.^20^^,^^22^^,^^25^^,^^37^^,^^59^ Furthermore, 1 study observed an increase in clinical preparedness and confidence from pretest to posttest and during a follow-up assessment 12 weeks later.^25^ Three studies reported a statistically significant increase in participants’ self-efficacy scores from pretest to posttest.^30^^,^^34^^,^^35^ Regarding comfort levels, 1 study reported statistically significant improvement from pretest to posttest,^20^ whereas 2 other studies reported no change.^24^^,^^33^ Table 2 provides an outcomes summary.

Two studies evaluated participants’ self-perceived knowledge about transgender HEADSS (home, education/employment, activities, drugs, sexuality, suicide/depression) assessments before and after an intervention.^23^^,^^30^ Another study assessed whether participants asked about suicidality in standardized patient encounters.^35^ Two studies incorporated their own knowledge-based question regarding suicidal thoughts, behavior, attempts, and risk in LGBTQ+ youth: “LGBT adolescents attempt suicide in the same proportions to those observed among heterosexual adolescents”^32^ and “LGBTQ youth are approximately 2 to 4 times as likely as non-LGBTQ youth to attempt suicide.”^26^ However, no study directly addressed the prevalence of suicidal ideation or attempts among LGBTQ+ youth following cultural competency training.

## Discussion

This systematic review evaluated studies on the effectiveness of LGBTQ+ cultural competency training for health care professionals regarding LGBTQ+ youth. The review synthesized 4 key aspects of cultural competency: knowledge, skills, attitudinal awareness, and behaviors. Studies on LGBTQ+ cultural competency training for health care workers showed variability in outcomes across domains such as knowledge, skills, attitudes, and comfort levels. Although many studies reported improvements, their sustainability was inconsistent, suggesting the need for ongoing reinforcement. It is important to explore factors contributing to sustaining these improvements and identify the best practices for long-term effectiveness.

Concerning knowledge, improvements were generally seen in both objective and self-perceived assessments. However, the lack of alignment suggests that self-assessments may not accurately reflect progress. Integrating both assessment types could provide a clearer view of training outcomes and ensure that self-perceived knowledge aligns with factual understanding. Cultural competency training improved skills and attitudinal awareness, but follow-up assessments showed challenges in maintaining these improvements over time.

Continuous reinforcement through periodic refreshers and ongoing education is important to preserve and build on initial improvements in health care providers’ attitudinal awareness and skills after culturally competent training regarding LGBTQ+ youth. Research is needed to determine the optimal frequency and format of reinforcement to sustain attitudinal changes and skills in health care providers. Only 1 study assessed behavioral changes,^37^ showing significant improvements, but more research is needed to explore how cultural competency training impacts health care practices and interactions with LGBTQ+ youth in patient care.

Furthermore, several studies assessed self-perceived competence domains, such as clinical preparedness/confidence, comfort levels, and self-efficacy. Although there were significant improvements in clinical preparedness/confidence and self-efficacy, the results regarding comfort levels were mixed. This variability suggests that whereas training can enhance many aspects of clinical practice, achieving consistent improvements in comfort levels may require additional strategies. Targeted approaches are needed to effectively address comfort levels in health care providers when caring for LGBTQ+ youth.

Most studies in our review relied exclusively on self-assessment measures. However, research suggests limited evidence of a robust correlation between self-assessment and actual abilities.^63^ Self-assessment can also introduce response-shift bias, where participants’ understanding of the measured construct changes over time.^64^ This bias can obscure the true impact on knowledge outcomes, especially in studies using only pretest and posttest designs,^65^ making it difficult to assess initial knowledge levels when participants are unfamiliar with the topic. Only 1 study in our review used a retrospective pretest/posttest study design, which can mitigate such bias. Future studies should incorporate retrospective pretest/posttest designs and objective measures alongside self-assessments to reduce bias, improve accuracy, and provide a clearer evaluation of health care professionals’ cultural competence.

Furthermore, changes in knowledge or attitudes do not necessarily predict changes in behavior or clinical practice.^66^ With the exception of 1 study^36^ measuring electronic medical record documentation practices, most studies in our review did not measure observable changes in clinical practice. To truly assess the effectiveness of LGBTQ+ cultural competency training, it is essential to also measure observable changes in behavior. Focusing solely on education and training overlooks institutional and systemic barriers that can hinder reforms to clinical practice. Future studies should incorporate assessments of observable changes in behavior (eg, asking for pronouns and rates of sexual orientation/gender identity documentation in electronic medical records) to understand how cultural competency improvements impact clinical practice.

Despite improvements in health care providers’ competencies, no studies directly linked LGBTQ+ youth cultural competency training to reductions in suicidal ideation or attempts among LGBTQ+ youth. This revealed a significant gap in the literature. Of the 19 studies, only 6 addressed clinicians’ knowledge of LGBTQ+ youth suicidal thoughts, behaviors, attempts, and risk.^23^^,^^26^^,^^30^^,^^32^^,^^34^^,^^35^ Still, these primarily addressed general LGBTQ+ youth cultural competency and did not explore the impact on suicide risk or behavioral changes. Given the rising rates of suicidal thoughts, behavior, attempts, and risk among LGBTQ+ youth,^52^^,^^53^ there is a pressing need for targeted training to enhance the ability of health care workers to recognize and intervene effectively in preventing suicide. Future research should explore not only the effectiveness of such training on health care professionals’ knowledge, but also its implications for patient outcomes, particularly in reducing suicidal ideation and attempts among LGBTQ+ youth, and focus on exploring the association between cultural competency training for health care providers and improved suicide prevention efforts for this population.

Recent research has increasingly focused on understanding and addressing the health care needs of transgender and nonbinary youth. These studies reflect a growing recognition of the disparities faced by these youth within health care settings, including limited access to gender-affirming care, disparities in mental health outcomes, and challenges in health care interactions.^19^^,^^60^^,^^67^ Of the 19 studies included in our analysis, 9 focused on transgender and nonbinary youth specifically.^23^^,^^28^, ^29^, ^30^, ^31^^,^^35^, ^36^, ^37^^,^^59^ Many of these studies highlighted the critical need for tailored interventions to effectively address the diverse health care needs of this population. This approach not only aims to improve health care access and outcomes, but also strives to create an inclusive health care environment where all youth, regardless of gender identity, feel understood and supported. Moving forward, there is a pressing need for ongoing research and the implementation of targeted training programs to address these health care disparities. This proactive approach will contribute to narrowing gaps and enhancing the health care experiences of transgender and nonbinary youth in health care settings.

Many studies focusing on youth-related LGBTQ+ cultural competency training among health care workers lacked follow-up measures. Only 4 of the 19 studies included in our analysis reported outcomes related to follow-up measures.^25^, ^26^, ^27^^,^^29^ This oversight limits our ability to determine whether initial improvements in outcome measures will persist over time. However, the few studies that included follow-up assessments provide valuable insight. These studies allow us to gauge the durability of these improvements and whether health care workers sustain their improved cultural competence in caring for LGBTQ+ youth. This understanding provides a comprehensive perspective of the effectiveness of the training in promoting lasting changes in health care workers’ approaches and interactions with LGBTQ+ youth. A comprehensive, long-term analysis is essential for evaluating the broader impact of LGBTQ+ cultural competency initiatives in health care settings. Further investigation is needed with longer follow-up periods and longitudinal designs to better understand the persistence of these improvements and their influence on long-term clinical outcomes for LGBTQ+ youth.

The included studies had several methodological limitations. Several studies in our review faced challenges with low response rates and high attrition rates, particularly in follow-up phases. Nonresponse and attrition raise concerns about study validity if the remaining participants differ systematically from those who drop out.^68^ This is relevant given our focus on health care professionals’ attitudes toward LGBTQ+ youth, which may vary between those participating in LGBTQ+ cultural competency training and those who do not. High attrition rates during follow-up could introduce the risk of bias, as only the highly motivated participants might complete the follow-up, potentially skewing retention outcomes to suggest greater effectiveness of the training programs. Importantly, none of the studies reviewed incorporated post hoc approaches to address the risk of biases from nonresponse and attrition. To enhance the validity of research, future studies should seek to reduce the impact of attrition through strategies such as incentives, improved participant engagement, and post hoc statistical methods.

Additionally, most studies lacked rigor in their designs, with inconsistent and unclear conceptualization for the phenomenon of interest, cultural competency, and its measurement. Less than half of the studies used validated measures grounded on theory and extensive research for cultural competency, yet these were solely based on self-reporting.^21^^,^^22^^,^^25^, ^26^, ^27^^,^^34^ Although common, self-report measurements may also introduce response shifts and increase the likelihood of statistical regression to the mean owing to inaccurate pretest ratings, potentially obscuring the true effects of the intervention.^69^ Of the 19 studies reviewed, only 6 used validated instruments such as the LGBT-DOCSS and SOCCS.^22^^,^^23^^,^^26^, ^27^, ^28^^,^^35^ Many studies used author-developed instruments that lacked formal validation, and only 2 studies provided reliability measures,^35^^,^^59^ such as Cronbach α, to assess the internal consistency of their tools. This raises concerns about the validity of their results and complicates comparisons between training programs. Adding to the challenge, there is considerable variation among the training programs in terms of format, duration, session numbers, and the specific topics covered. Although these differences partly reflect the diverse disciplines involved in the interventions, we recommend that future studies incorporate standardized, validated instruments to ensure greater consistency in training protocols. This would improve the comparability of findings and strengthen the conclusions drawn from such interventions.

Another methodological challenge was the limited use of RCTs. The only RCT included had a strong design,^21^ featuring convenience sampling, sensitivity analysis, effect size, low attrition, and a widely accepted training manual. However, it had limitations including a pretest/posttest design with no follow-ups and insufficient detail on randomization procedures, raising questions regarding whether enrollment was independent of the principal investigator before participant enrollment or if double blinding was used. To address these issues, studies would benefit from using validated instruments, follow-up assessments, and more rigorous experimental designs, including clearer randomization and stronger methodological controls.

Our study has a key limitation: it includes only English-language publications, excluding potentially valuable non-English research, especially from regions such as Latin America and the Caribbean, Asia, and other continents globally.^70^^,^^71^ Including such studies could enhance the global understanding of LGBTQ+ health care interventions for adolescents. Additionally, excluding non-English studies also restricted the applicability of our findings across various cultural contexts.

In summary, although LGBTQ+ youth cultural competency training improves objective measures of knowledge, skills, and clinical preparedness, the improvements in self-perceived knowledge, attitudes, and comfort levels are inconsistent. Importantly, there is a significant research gap in the current literature about how such training affects suicide prevention outcomes among LGBTQ+ youth. Given the critical need for addressing mental health and suicide risk in LGBTQ+ youth, future research should focus on examining the association between health care provider cultural competency training and suicidal risk in the LGBTQ+ youth population. Future research should integrate self-assessment with objective evaluations to improve training effectiveness. Longitudinal studies are crucial for understanding the sustained impact on clinical practices and patient outcomes. Additionally, employing standardized, validated assessment tools will enhance the reliability of results and facilitate comparisons between training programs. Investigating new training methods and strategies to enhance comfort of health care professionals and overcome systemic barriers is also essential. These efforts will help refine training programs and ultimately improve the quality of care for LGBTQ+ youth and advance health care equity.

## CRediT authorship contribution statement

**Kelly Cembrale:** Writing – original draft, Project administration, Investigation, Data curation, Writing – review & editing, Visualization, Methodology, Formal analysis, Conceptualization. **Ioannis Demopoulos:** Writing – review & editing, Methodology, Formal analysis, Conceptualization, Writing – original draft, Investigation, Data curation. **Angelica Terepka:** Writing – review & editing, Supervision, Investigation, Data curation, Writing – original draft, Methodology, Formal analysis, Conceptualization. **Leonell Torres-Pagán:** Writing – original draft, Methodology, Formal analysis, Writing – review & editing, Supervision, Investigation, Data curation. **John Usseglio:** Validation, Software, Data curation, Supervision, Methodology.
