# Supplementary material for: Systematic Review: Exploring the Effectiveness of Health Care Competency Training as a Means of Suicide Prevention in LGBTQ+ Youth
Source: JAACAP Open. 2025 Jul 7;4(1):8–28. doi: 10.1016/j.jaacop.2025.06.010 (PMC12925907; doi:10.1016/j.jaacop.2025.06.010)
Supplement: Supplementary Table S1 [file mmc1.pdf]

Table S1 Search algorithms.

Databases	Search Algorithms
Database: APA PsycInfo<br>Platform: EBSCO<br>Searched on: 5/20/2024<br>Results: 1,199	
	(DE "Adolescent Health" OR DE "Early Adolescence" OR DE "Late Adolescence" OR Adolescen* OR Teen* OR “Young adult*” OR “Young people*” OR “Young person*” OR Youth*) AND (DE "Gender Identity" OR DE "Gender Nonbinary" OR DE "Gender Nonconforming" OR DE "LGBTQ" OR DE "Two-Spirit" OR DE "Homosexuality" OR DE "Lesbianism" OR DE "Male Homosexuality" OR DE "Intersex" OR DE "Sexual Orientation" OR DE "Asexuality" OR DE "Pansexuality" OR DE "Sexual Minority Groups" OR DE "Transgender" OR Asexual* OR Bisexual* OR Gay OR Gays OR “Gender-divers*” OR “Gender-fluid*” OR (Gender N5 identit*) OR (Gender N5 minorit*) OR “Gender-nonconforming” OR “Gender non-conforming” OR Genderqueer* OR “Gender-queer*” OR “GLBT*” OR Homosexual* OR Intersex* OR “LBG” OR “LBGT*” OR Lesbian* OR Lesbigay* OR “LGBT*” OR “Men who have sex with men” OR “MSM” OR “Non-heterosexual*” OR Nonbinary OR “Non-binary” OR Pansexual* OR Queer* OR (Sexual N5 minorit*) OR (Sexual N5 orientation*) OR “Trans-gender*” OR “Trans-feminine*” OR “Trans-Man” OR “Trans-masculine” OR “Trans-Men” OR “Trans-Woman” OR “Trans-Women” OR Transfeminine* OR Transgender* OR Transmasculine* OR Transexual* OR Transsexual* OR “Two-spirit people*” OR “Two-spirit person*” OR “Women who have sex with women” OR “WSW”) AND (DE "Health Personnel" OR DE "Allied Health Personnel" OR DE "Medical Personnel" OR DE "Mental Health Personnel" OR DE "Nurses" OR DE "Physicians" OR (Health N5 personnel) OR (healthcare N5 personnel) OR (health N5 professional*) OR (healthcare N5 professional*) OR (health N5 worker*) OR (healthcare N5 worker*) OR (medical N5 personnel) OR doctor* OR physician* OR clinician* OR provider* OR fellow* OR resident* OR intern OR interns OR counselor* OR nurs* OR “nurse practitioner*” OR “DNP” OR staff OR student* OR trainee*) AND (DE "Cultural Competence" OR DE "Professional Competence" OR DE "Professionalism" OR DE "Gender Affirming Care" OR DE "Gender Reassignment" OR DE "Hormone Therapy" OR DE "Health Care Delivery" OR DE "Clinical Practice" OR DE "Managed Care" OR (Adequate N5 care) OR (Adequate N5 healthcare) OR (Affirming N5 care) OR (Affirming N5 healthcare) OR Competen* OR “Cross-cultur*” OR “Cross-cultural care” OR “Cross-cultural service*” OR (Cultural N5 care) OR (Cultural N5 service*) OR (Cultural N5 competenc*) OR (Cultural N5 humility) OR (Cultural N5 sensitivity) OR (Culturally N5 competent) OR “Culturally-competent care” OR “Culturally-competent service*” OR (Culturally N5 congruent) OR “Culturally-congruent care” OR “Culturally-congruent service*” OR (Culturally N5 sensitive) OR “Culturally-sensitive care” OR “Culturally-sensitive service*” OR (Care N5 equalit*) OR (Care N5 equit*) OR (Care N5 inequalit*) OR (Care N5 inequit*) OR (Deliver N5 care) OR (Deliver N5 healthcare) OR (Deliver N5 service*) OR (Delivery N5 care) OR (Delivery N5 healthcare) OR (Delivery N5 service*) OR (Gender N5 affirm*) OR (Gender N5 confirm*) OR (Gender N5 conform*) OR (Provide N5 care) OR (Provide N5 healthcare) OR (Provide N5 service*) OR (Quality N5 care) OR (Quality N5 healthcare) OR “Self-effica*” OR “transcultural care” OR “trans-cultural care”) AND (DE "Education" OR DE "Curriculum" OR DE "Educational Programs" OR DE "Personnel Training" OR DE "Quality of Services" OR DE "Quality of Care" OR DE "Quality Control" OR Curricul* OR Develop* OR Educat* OR Evaluat* OR Initiative* OR Intervention* OR Learn* OR Module* OR Program* OR Project* OR Prepare* OR (Quality N5 improve*) OR “Standardized patient*” OR Train*)
Database: Cumulated Index in Nursing and Allied Health Literature (CINAHL)<br>Platform: EBSCO<br>Searched on: 5/20/2024<br>Results: 972	
	((MH "Adolescence+") OR (MH "Young Adult") OR Adolescen* OR Teen* OR “Young adult*” OR “Young people*” OR “Young person*” OR Youth*) AND ((MH "Asexuality") OR (MH "Bisexuality") OR (MH "Gender Identity+") OR (MH "Nonbinary Persons") OR (MH "Homosexuality") OR (MH "Intersex Persons") OR (MH "Sexual and Gender Minorities+") OR (MH "Sexual Orientation+") OR (MH "Transgender Persons+") OR Asexual* OR Bisexual* OR Gay OR Gays OR “Gender-divers*” OR “Gender-fluid*” OR (Gender N5 identit*) OR (Gender N5 minorit*) OR “Gender-nonconforming” OR “Gender non-conforming” OR Genderqueer* OR “Gender-queer*” OR “GLBT*” OR Homosexual* OR Intersex* OR “LBG” OR “LBGT*” OR Lesbian* OR Lesbigay* OR “LGBT*” OR “Men who have sex with men” OR “MSM” OR “Non-heterosexual*” OR Nonbinary OR “Non-binary” OR Pansexual* OR Queer* OR (Sexual N5 minorit*) OR (Sexual N5 orientation*) OR “Trans-gender*” OR “Trans-feminine*” OR “Trans-Man” OR “Trans-masculine” OR “Trans-Men” OR “Trans-Woman” OR “Trans-Women” OR Transfeminine* OR Transgender* OR Transmasculine* OR Transexual* OR Transsexual* OR “Two-spirit people*” OR “Two-spirit person*” OR “Women who have sex with women” OR “WSW”) AND ((MH "Health Personnel+") OR (Health N5 personnel) OR (healthcare N5 personnel) OR (health N5 professional*) OR (healthcare N5 professional*) OR (health N5 worker*) OR (healthcare N5 worker*) OR (medical N5 personnel) OR doctor* OR physician* OR clinician* OR provider* OR fellow* OR resident* OR intern OR interns OR counselor* OR nurs* OR “nurse practitioner*” OR “DNP” OR staff OR student* OR trainee*) AND ((MH "Clinical Competence+") OR (MH "Gender Affirming Care+") OR (MH "Health Care Delivery+") OR (MH "Transcultural Care") OR (Adequate N5 care) OR (Adequate N5 healthcare) OR (Affirming N5 care) OR (Affirming N5 healthcare) OR Competen* OR “Cross-cultur*” OR “Cross-cultural care” OR “Cross-cultural service*” OR (Cultural N5 care) OR (Cultural N5 service*) OR (Cultural N5 competenc*) OR (Cultural N5 humility) OR (Cultural N5 sensitivity) OR (Culturally N5 competent) OR “Culturally-competent care” OR “Culturally-competent service*” OR (Culturally N5 congruent) OR “Culturally-congruent care” OR “Culturally-congruent service*” OR (Culturally N5 sensitive) OR “Culturally-sensitive care” OR “Culturally-sensitive service*” OR (Care N5 equalit*) OR (Care N5 equit*) OR (Care N5 inequalit*) OR (Care N5 inequit*) OR (Deliver N5 care) OR (Deliver N5 healthcare) OR (Deliver N5 service*) OR (Delivery N5 care) OR (Delivery N5 healthcare) OR (Delivery N5 service*) OR (Gender N5 affirm*) OR (Gender N5 confirm*) OR (Gender N5 conform*) OR (Provide N5 care) OR (Provide N5 healthcare) OR (Provide N5 service*) OR (Quality N5 care) OR (Quality N5 healthcare) OR “Self-effica*” OR “transcultural care” OR “trans-cultural care”) AND ((MH "Education+") OR (MH "Quality Improvement+") OR Curricul* OR Develop* OR Educat* OR Evaluat* OR Initiative* OR Intervention* OR Learn* OR Module* OR Program* OR Project* OR Prepare* OR (Quality N5 improve*) OR “Standardized patient*” OR Train*)

Database: Embase
Platform: Elsevier
Searched on: 5/20/2024
Results: 2,035

('adolescent'/exp OR 'young adult'/exp OR Adolescen*:ti,ab,kw OR Teen*:ti,ab,kw OR ‘Young adult*’:ti,ab,kw OR ‘Young people*’:ti,ab,kw OR ‘Young person*’:ti,ab,kw OR Youth*:ti,ab,kw) AND ('asexuality'/exp OR 'bisexuality'/exp OR 'gender identity'/exp OR 'gender nonbinary'/exp OR 'homosexuality'/exp OR 'intersex'/exp OR 'sexual and gender minority'/exp OR 'sexual orientation'/exp OR 'transgender'/exp OR Asexual*:ti,ab,kw OR Bisexual*:ti,ab,kw OR Gay:ti,ab,kw OR Gays:ti,ab,kw OR ‘Gender-divers*’:ti,ab,kw OR ‘Gender-fluid*’:ti,ab,kw OR ((Gender NEAR/5 identit*):ti,ab,kw) OR ((Gender NEAR/5 minorit*):ti,ab,kw) OR ‘Gender-nonconforming’:ti,ab,kw OR ‘Gender non-conforming’:ti,ab,kw OR Genderqueer*:ti,ab,kw OR ‘Gender-queer*’:ti,ab,kw OR ‘GLBT*’:ti,ab,kw OR Homosexual*:ti,ab,kw OR Intersex*:ti,ab,kw OR ‘LBG’:ti,ab,kw OR ‘LBGT*’:ti,ab,kw OR Lesbian*:ti,ab,kw OR Lesbigay*:ti,ab,kw OR ‘LGBT*’:ti,ab,kw OR ‘Men who have sex with men’:ti,ab,kw OR ‘MSM’:ti,ab,kw OR ‘Non-heterosexual*’:ti,ab,kw OR Nonbinary:ti,ab,kw OR ‘Non-binary’:ti,ab,kw OR Pansexual*:ti,ab,kw OR Queer*:ti,ab,kw OR ((Sexual NEAR/5 minorit*):ti,ab,kw) OR ((Sexual NEAR/5 orientation*):ti,ab,kw) OR ‘Trans-gender*’:ti,ab,kw OR ‘Trans-feminine*’:ti,ab,kw OR ‘Trans-Man’:ti,ab,kw OR ‘Trans-masculine’:ti,ab,kw OR ‘Trans-Men’:ti,ab,kw OR ‘Trans-Woman’:ti,ab,kw OR ‘Trans-Women’:ti,ab,kw OR Transfeminine*:ti,ab,kw OR Transgender*:ti,ab,kw OR Transmasculine*:ti,ab,kw OR Transexual*:ti,ab,kw OR Transsexual*:ti,ab,kw OR ‘Two-spirit people*’:ti,ab,kw OR ‘Two-spirit person*’:ti,ab,kw OR ‘Women who have sex with women’:ti,ab,kw OR ‘WSW’:ti,ab,kw) AND ('health care personnel'/exp OR 'nurse'/exp OR ((Health NEAR/5 personnel):ti,ab,kw) OR ((healthcare NEAR/5 personnel):ti,ab,kw) OR ((health NEAR/5 professional*):ti,ab,kw) OR ((healthcare NEAR/5 professional*):ti,ab,kw) OR ((health NEAR/5 worker*):ti,ab,kw) OR ((healthcare NEAR/5 worker*):ti,ab,kw) OR ((medical NEAR/5 personnel):ti,ab,kw) OR doctor*:ti,ab,kw OR physician*:ti,ab,kw OR clinician*:ti,ab,kw OR provider*:ti,ab,kw OR fellow*:ti,ab,kw OR resident*:ti,ab,kw OR intern:ti,ab,kw OR interns:ti,ab,kw OR counselor*:ti,ab,kw OR nurs*:ti,ab,kw OR ‘nurse practitioner*’:ti,ab,kw OR ‘DNP’:ti,ab,kw OR staff:ti,ab,kw OR student*:ti,ab,kw OR trainee*:ti,ab,kw) AND ('clinical competence'/exp OR 'gender-affirming care'/exp OR 'health care delivery'/exp OR 'transcultural care'/exp OR ((Adequate NEAR/5 care):ti,ab,kw) OR ((Adequate NEAR/5 healthcare):ti,ab,kw) OR ((Affirming NEAR/5 care):ti,ab,kw) OR ((Affirming NEAR/5 healthcare):ti,ab,kw) OR Competen*:ti,ab,kw OR ‘Cross-cultur*’:ti,ab,kw OR ‘Cross-cultural care’:ti,ab,kw OR ‘Cross-cultural service*’:ti,ab,kw OR ((Cultural NEAR/5 care):ti,ab,kw) OR ((Cultural NEAR/5 service*):ti,ab,kw) OR ((Cultural NEAR/5 competenc*):ti,ab,kw) OR ((Cultural NEAR/5 humility):ti,ab,kw) OR ((Cultural NEAR/5 sensitivity):ti,ab,kw) OR ((Culturally NEAR/5 competent):ti,ab,kw) OR ‘Culturally-competent care’:ti,ab,kw OR ‘Culturally-competent service*’:ti,ab,kw OR ((Culturally NEAR/5 congruent):ti,ab,kw) OR ‘Culturally-congruent care’:ti,ab,kw OR ‘Culturally-congruent service*’:ti,ab,kw OR ((Culturally NEAR/5 sensitive):ti,ab,kw) OR ‘Culturally-sensitive care’:ti,ab,kw OR ‘Culturally-sensitive service*’:ti,ab,kw OR ((Care NEAR/5 equalit*):ti,ab,kw) OR ((Care NEAR/5 equit*):ti,ab,kw) OR ((Care NEAR/5 inequalit*):ti,ab,kw) OR ((Care NEAR/5 inequit*):ti,ab,kw) OR ((Deliver NEAR/5 care):ti,ab,kw) OR ((Deliver NEAR/5 healthcare):ti,ab,kw) OR ((Deliver NEAR/5 service*):ti,ab,kw) OR ((Delivery NEAR/5 care):ti,ab,kw) OR ((Delivery NEAR/5 healthcare):ti,ab,kw) OR ((Delivery NEAR/5 service*):ti,ab,kw) OR ((Gender NEAR/5 affirm*):ti,ab,kw) OR ((Gender NEAR/5 confirm*):ti,ab,kw) OR ((Gender NEAR/5 conform*):ti,ab,kw) OR ((Provide NEAR/5 care):ti,ab,kw) OR ((Provide NEAR/5 healthcare):ti,ab,kw) OR ((Provide NEAR/5 service*):ti,ab,kw) OR ((Quality NEAR/5 care):ti,ab,kw) OR ((Quality NEAR/5 healthcare):ti,ab,kw) OR ‘Self-effica*’:ti,ab,kw OR ‘transcultural care’:ti,ab,kw OR ‘trans-cultural care’:ti,ab,kw) AND ('education'/exp OR 'total quality management'/exp OR Curricul*:ti,ab,kw OR Develop*:ti,ab,kw OR Educat*:ti,ab,kw OR Evaluat*:ti,ab,kw OR Initiative*:ti,ab,kw OR Intervention*:ti,ab,kw OR Learn*:ti,ab,kw OR Module*:ti,ab,kw OR Program*:ti,ab,kw OR Project*:ti,ab,kw OR Prepare*:ti,ab,kw OR ((Quality NEAR/5 improve*):ti,ab,kw) OR ‘Standardized patient*’:ti,ab,kw OR Train*:ti,ab,kw)

Database: ProQuest Dissertations & Theses Global
Platform: ProQuest
Searched on: 5/20/2024
Results: 219

noft((Adolescen* OR Teen* OR “Young adult*” OR “Young people*” OR “Young person*” OR Youth*) AND (Asexual* OR Bisexual* OR Gay OR Gays OR “Gender-divers*” OR “Gender-fluid*” OR (Gender NEAR/5 identit*) OR (Gender NEAR/5 minorit*) OR “Gender-nonconforming” OR “Gender non-conforming” OR Genderqueer* OR “Gender-queer*” OR “GLBT*” OR Homosexual* OR Intersex* OR “LBG” OR “LBGT*” OR Lesbian* OR Lesbigay* OR “LGBT*” OR “Men who have sex with men” OR “MSM” OR “Non-heterosexual*” OR Nonbinary OR “Non-binary” OR Pansexual* OR Queer* OR (Sexual NEAR/5 minorit*) OR (Sexual NEAR/5 orientation*) OR “Trans-gender*” OR “Trans-feminine*” OR “Trans-Man” OR “Trans-masculine” OR “Trans-Men” OR “Trans-Woman” OR “Trans-Women” OR Transfeminine* OR Transgender* OR Transmasculine* OR Transexual* OR Transsexual* OR “Two-spirit people*” OR “Two-spirit person*” OR “Women who have sex with women” OR “WSW”) AND ((Health NEAR/5 personnel) OR (healthcare NEAR/5 personnel) OR (health NEAR/5 professional*) OR (healthcare NEAR/5 professional*) OR (health NEAR/5 worker*) OR (healthcare NEAR/5 worker*) OR (medical NEAR/5 personnel) OR doctor* OR physician* OR clinician* OR provider* OR fellow* OR resident* OR intern OR interns OR counselor* OR nurs* OR “nurse practitioner*” OR “DNP” OR staff OR student* OR trainee*) AND ((Adequate NEAR/5 care) OR (Adequate NEAR/5 healthcare) OR (Affirming NEAR/5 care) OR (Affirming NEAR/5 healthcare) OR Competen* OR “Cross-cultur*” OR “Cross-cultural care” OR “Cross-cultural service*” OR (Cultural NEAR/5 care) OR (Cultural NEAR/5 service*) OR (Cultural NEAR/5 competenc*) OR (Cultural NEAR/5 humility) OR (Cultural NEAR/5 sensitivity) OR (Culturally NEAR/5 competent) OR “Culturally-competent care” OR “Culturally-competent service*” OR (Culturally NEAR/5 congruent) OR “Culturally-congruent care” OR “Culturally-congruent service*” OR (Culturally NEAR/5 sensitive) OR “Culturally-sensitive care” OR “Culturally-sensitive service*” OR (Care NEAR/5 equalit*) OR (Care NEAR/5 equit*) OR (Care NEAR/5 inequalit*) OR (Care NEAR/5 inequit*) OR (Deliver NEAR/5 care) OR (Deliver NEAR/5 healthcare) OR (Deliver NEAR/5 service*) OR (Delivery NEAR/5 care) OR (Delivery NEAR/5 healthcare) OR (Delivery NEAR/5 service*) OR (Gender NEAR/5 affirm*) OR (Gender NEAR/5 confirm*) OR (Gender NEAR/5 conform*) OR (Provide NEAR/5 care) OR (Provide NEAR/5 healthcare) OR (Provide NEAR/5 service*) OR (Quality NEAR/5 care) OR (Quality NEAR/5 healthcare) OR “Self-effica*” OR “transcultural care” OR “trans-cultural care”) AND (Curricul* OR Develop* OR Educat* OR Evaluat* OR Initiative* OR Intervention* OR Learn* OR Module* OR Program* OR Project* OR Prepare* OR (Quality NEAR/5 improve*) OR “Standardized patient*” OR Train*))

Database: PubMed
Platform: NCBI
Searched on: 5/20/2024
Results: 1,776

("Adolescent"[MeSH] OR "Young Adult"[MeSH] OR Adolescen*[tiab] OR Teen*[tiab] OR “Young adult*”[tiab] OR “Young people*”[tiab] OR “Young person*”[tiab] OR Youth*[tiab]) AND ("Bisexuality"[MeSH] OR "Gender Identity"[MeSH] OR "Gender-Nonconforming Persons"[MeSH] OR "Homosexuality"[MeSH] OR "Intersex Persons"[MeSH] OR "Sexual and Gender Minorities"[MeSH] OR "Transgender Persons"[MeSH] OR Asexual*[tiab] OR Bisexual*[tiab] OR Gay[tiab] OR Gays[tiab] OR “Gender-divers*”[tiab] OR “Gender-fluid*”[tiab] OR “Gender identity”[tiab:~5] OR “Gender identities”[tiab:~5] OR “Gender minority”[tiab:~5] OR “Gender minorities”[tiab:~5] OR “Gender-nonconforming”[tiab] OR “Gender non-conforming”[tiab] OR Genderqueer*[tiab] OR “Gender-queer*”[tiab] OR “GLBT*”[tiab] OR Homosexual*[tiab] OR Intersex*[tiab] OR “LBG”[tiab] OR “LBGT*”[tiab] OR Lesbian*[tiab] OR Lesbigay*[tiab] OR “LGBT*”[tiab] OR “Men who have sex with men”[tiab] OR “MSM”[tiab] OR “Non-heterosexual*”[tiab] OR Nonbinary[tiab] OR “Non-binary”[tiab] OR Pansexual*[tiab] OR Queer*[tiab] OR “Sexual minority”[tiab:~5] OR “Sexual minorities”[tiab:~5] OR “Sexual orientation”[tiab:~5] OR “Sexual orientations”[tiab:~5] OR “Trans-gender*”[tiab] OR “Trans-feminine*”[tiab] OR “Trans-Man”[tiab] OR “Trans-masculine”[tiab] OR “Trans-Men”[tiab] OR “Trans-Woman”[tiab] OR “Trans-Women”[tiab] OR Transfeminine*[tiab] OR Transgender*[tiab] OR Transmasculine*[tiab] OR Transexual*[tiab] OR Transsexual*[tiab] OR “Two-spirit people*”[tiab] OR “Two-spirit person*”[tiab] OR “Women who have sex with women”[tiab] OR “WSW”[tiab]) AND ("Health Personnel"[Mesh] OR “Health personnel”[tiab:~5] OR “healthcare personnel”[tiab:~5] OR “health professional”[tiab:~5] OR “health professionals”[tiab:~5] OR “healthcare professional”[tiab:~5] OR “healthcare professionals”[tiab:~5] OR “health worker”[tiab:~5] OR “health workers”[tiab:~5] OR “healthcare worker”[tiab:~5] OR “healthcare workers”[tiab:~5] OR “medical personnel”[tiab:~5] OR doctor*[tiab] OR physician*[tiab] OR clinician*[tiab] OR provider*[tiab] OR fellow*[tiab] OR resident*[tiab] OR intern[tiab] OR interns[tiab] OR counselor*[tiab] OR nurs*[tiab] OR “nurse practitioner*”[tiab] OR “DNP”[tiab] OR staff[tiab] OR student*[tiab] OR trainee*[tiab]) AND ("Clinical Competence"[MeSH] OR "Culturally Competent Care"[MeSH] OR "Delivery of Health Care"[MeSH] OR "Gender-Affirming Care"[Mesh] OR “Adequate care”[tiab:~5] OR “Adequate healthcare”[tiab:~5] OR “Affirming care”[tiab:~5] OR “Affirming healthcare”[tiab:~5] OR Competen*[tiab] OR “Cross-cultur*”[tiab] OR “Cross-cultural care”[tiab] OR “Cross-cultural service*”[tiab] OR “Cultural care”[tiab:~5] OR “Cultural service”[tiab:~5] OR “Cultural services”[tiab:~5] OR “Cultural competence”[tiab:~5] OR “Cultural competency”[tiab:~5] OR “Cultural competencies”[tiab:~5] OR “Cultural humility”[tiab:~5] OR “Cultural sensitivity”[tiab:~5] OR “Culturally competent”[tiab:~5] OR “Culturally-competent care”[tiab] OR “Culturally-competent service*”[tiab] OR “Culturally congruent”[tiab:~5] OR “Culturally-congruent care”[tiab] OR “Culturally-congruent service*”[tiab] OR “Culturally sensitive”[tiab:~5] OR “Culturally-sensitive care”[tiab] OR “Culturally-sensitive service*”[tiab] OR “Care equality”[tiab:~5] OR “Care equalities”[tiab:~5] OR “Care equity”[tiab:~5] OR “Care equities”[tiab:~5] OR “Care inequality”[tiab:~5] OR “Care inequalities”[tiab:~5] OR “Care inequity”[tiab:~5] OR “Care inequities”[tiab:~5] OR “Deliver care”[tiab:~5] OR “Deliver healthcare”[tiab:~5] OR “Deliver service”[tiab:~5] OR “Deliver services”[tiab:~5] OR “Delivery care”[tiab:~5] OR “Delivery healthcare”[tiab:~5] OR “Delivery service”[tiab:~5] OR “Delivery services”[tiab:~5] OR “Gender affirmative”[tiab:~5] OR “Gender affirming”[tiab:~5] OR “Gender confirmative”[tiab:~5] OR “Gender confirming”[tiab:~5] OR “Gender conformative”[tiab:~5] OR “Gender conforming”[tiab:~5] OR “Provide care”[tiab:~5] OR “Provide healthcare”[tiab:~5] OR “Provide service”[tiab:~5] OR “Provide services”[tiab:~5] OR “Quality care”[tiab:~5] OR “Quality healthcare”[tiab:~5] OR “Self-effica*”[tiab] OR “transcultural care”[tiab] OR “trans-cultural care”[tiab]) AND ("Education"[Mesh] OR "Health Personnel/education"[MeSH] OR "Quality Improvement"[Mesh] OR "Staff Development"[Mesh] OR Curricul*[tiab] OR Develop*[tiab] OR Educat*[tiab] OR Evaluat*[tiab] OR Initiative*[tiab] OR Intervention*[tiab] OR Learn*[tiab] OR Module*[tiab] OR Program*[tiab] OR Project*[tiab] OR Prepare*[tiab] OR “Quality Improve”[tiab:~5] OR “Quality improvement”[tiab:~5] OR “Standardized patient*”[tiab] OR Train*[tiab])

Database: Cochrane Central Register of Controlled Trials (CENTRAL)
Platform: Cochrane Library
Searched on: 5/20/2024
Results: 116

ID	Search	Hits
#1	MeSH descriptor: [Adolescent] explode all trees	136839
#2	MeSH descriptor: [Young Adult] explode all trees	95606
#3	(Adolescen* OR Teen* OR (Young NEXT adult*) OR (Young NEXT people*) OR (Young NEXT person*) OR Youth*):ti,ab,kw (Word variations have been searched)	243680
#4	#1 OR #2 OR #3	243680
#5	MeSH descriptor: [undefined] explode all trees	0
#6	MeSH descriptor: [Gender Identity] explode all trees	417
#7	MeSH descriptor: [Gender-Nonconforming Persons] explode all trees	0
#8	MeSH descriptor: [Homosexuality] explode all trees	923
#9	MeSH descriptor: [Intersex Persons] explode all trees	0
#10	MeSH descriptor: [Sexual and Gender Minorities] explode all trees	552
#11	MeSH descriptor: [Transgender Persons] explode all trees	145

Grey Literature Source: ClinicalTrials.gov
Platform: clinicaltrials.gov
Searched on: 5/20/2024
Results: 75

Other terms: LGBTQ OR LGBT Health OR Sexual and Gender Minorities OR Gender Identity OR Sexual Orientation OR Transgender
Intervention/treatment: transcultural care OR Health care delivery OR cultural competence training OR cultural competence OR cultural competency
Study Status: All Studies

Grey Literature Source: World Health Organization International Clinical Trials Registry Platform (WHO ICTRP)
Platform: who.int/clinical-trials-registry-platform
Searched on: 5/20/2024
Results: 2

(LGBTQ OR LGBT Health OR Sexual and Gender Minorities OR Gender Identity OR Sexual Orientation OR Transgender) AND (transcultural care OR Health care delivery OR cultural competence training OR cultural competence OR cultural competency)
